# The Impact of COVID-19 on Diverse Older Adults and Health Equity in the United States

**DOI:** 10.3389/fpubh.2021.661592

**Published:** 2021-05-17

**Authors:** Lourdes R. Guerrero, Steven P. Wallace

**Affiliations:** ^1^David Geffen School of Medicine at UCLA, Los Angeles, CA, United States; ^2^UCLA Fielding School of Public Health and Associate Director, UCLA Center for Health Policy Research, Los Angeles, CA, United States

**Keywords:** COVID-19, health equity, risk factors, African American, Latinx, older adult, social determinants

## Abstract

Older adults are most at risk of negative COVID-19 outcomes and consequences. This study applies the World Health Organization's Health Inequity Causal Model to identify different factors that may be driving the higher observed hospitalizations and deaths among older adults of color compared to non-Latinx Whites in the United States. We used multiple data sets, including the US Census American Community Survey and PULSE COVID data, along with published reports, to understand the social context of older adults, including income distributions by race and ethnicity, household composition and potential COVID-19 exposure to older adults by working family members. Our findings point to multiple social determinants of health, beyond individual health risks, which may explain why older adults of color are the most at risk of negative COVID-19 outcomes and consequences. Current health policies do not adequately address disproportionate impact; some even worsen it. This manuscript provides new data and analysis to support the call for equity-focused solutions to this pandemic and health in general in the future, focusing on meeting the needs of our most vulnerable communities.

## Introduction

The cases of COVID-19 in the United States have varied over time and between states since the beginning of the pandemic, but it has become apparent that rates of hospitalization and deaths are disproportionately affecting adults age 65 and over in communities of color. In a cross-sectional analysis of stay-at-home orders, COVID-19 cases and proportion of African American population in a state, researchers found that, overall, expected cumulative cases were reduced by the stay-at-home orders, yet number of cases and fatality rates were higher among the African American population (1). Similarly, another analysis of state-level data found that states with higher income inequality had a higher number of deaths of COVID-19 (2). According the Centers for Disease Control and Prevention (CDC), age-adjusted hospitalization dates for COVID-19 were 3.4 times higher for Latinx individuals, 3.3 higher for American Indian/Alaska Natives (AI/AN), and 3.0 times higher for Blacks than for non-Latinx Whites (3). The systematic differences that follow dimensions of social inequality suggest that social factors, beyond individual health risks, are likely to be driving inequities in the outcomes of the pandemic for older adults.

Selden and Berdahl (4) found that job characteristics and household composition were the factors that contributed to disparities in severe COVID-19 illness among Blacks and Latinxs. The pandemic has made it clear that where, and with whom, one lives affects health (e.g., the situation in long-term care facilities) and that many of these disparities have been longstanding (5, 6). The virus poses a specific threat to the lives emotional well-being of older adults, and even more so among persons of color, aged 65 and over (7).

**Figure 1:**
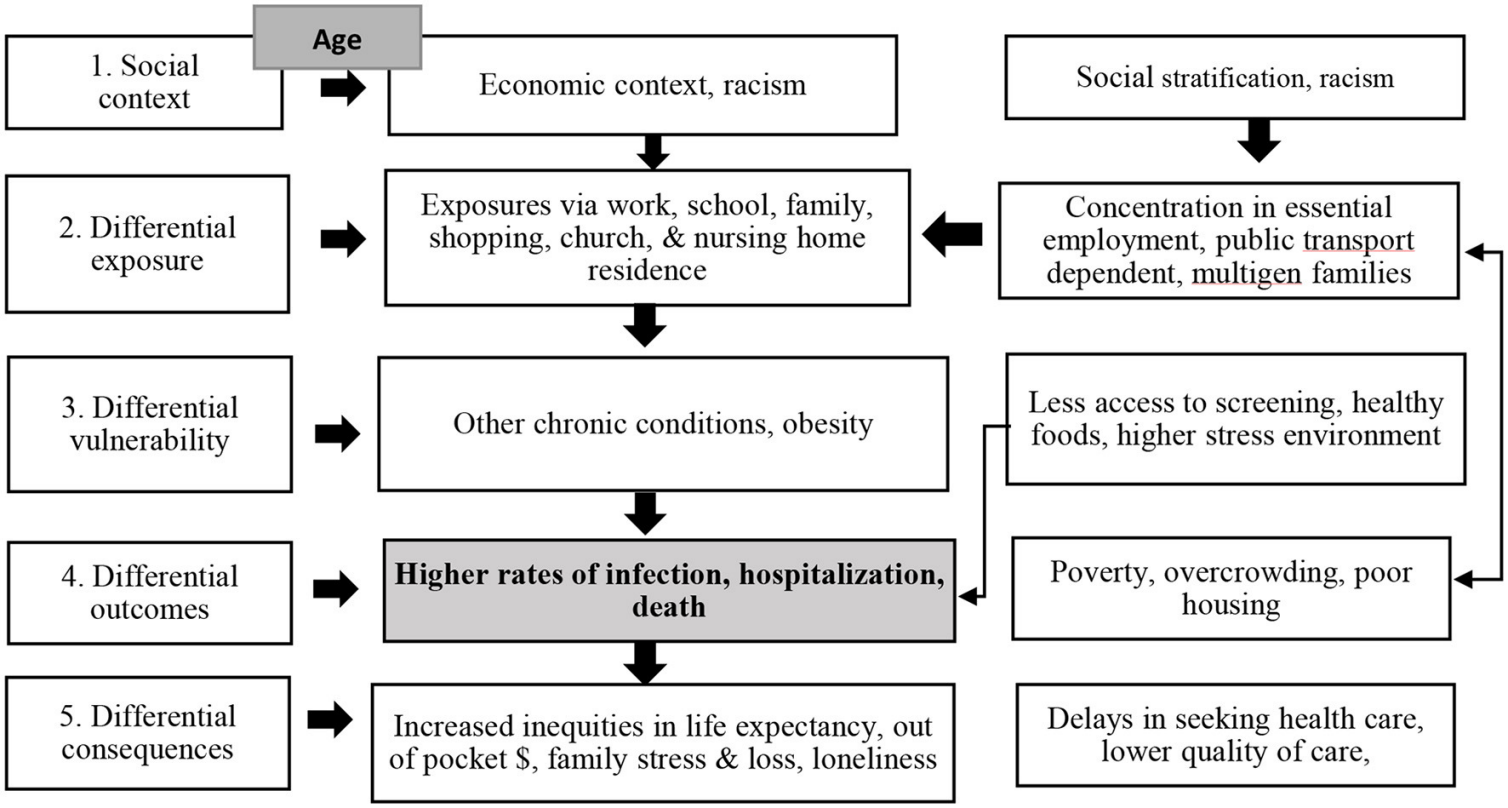
Health inequity causal model.

To identify the many different potential factors driving the higher observed hospitalizations and deaths among older adults of color compared to non-Latinx Whites, we apply the World Health Organization's Health Inequity Causal Model (8). The strength of this model is that it includes multiple dimensions of interacting and intersecting causes of health inequities, creating a more complex view of health inequities than a simple risk model (see Figure 1). The model starts at the social context level, a level which leads to a cascade of more proximal differential and inequitable impacts. Most relevant to COVID in older adults of color are the economic context of the US and communities of color, along with systemic historical racism (Level 1). We have also added the social consequences of age, which has consequences both in terms of the cumulative impact of poverty and racism, but also social expectations and policies that focus on older age groups.

As a highly contagious, primarily airborne, virus, older adults of color may have differential exposures compared to non-Latinx White older adults. Their pre-existing conditions and cumulative health disadvantages are likely to put them at increased vulnerability to those exposures. The resulting infections are likely to have differential outcomes in terms of morbidity, mortality, and health care use. And the consequences of the pandemic for older adults of color and their families may be worse than for non-Latinx Whites. By providing data at each step of this model we will show how older adults of color suffer inequities due to multiple mechanisms and in multiple ways, leading to a cascade of inequities that requires more than a single intervention to address. This analysis also highlights the consequences of systemic social and economic inequities on the health of older adults of color that has implications beyond the COVID-19 pandemic.

## Materials and Methods

We conducted a cross-sectional study using multiple data sets, including the US Census American Community Survey (9), US Census PULSE COVID survey (10) and the National Health Interview Survey (11), along with published reports to understand the social context of older adults, including income distributions by race and ethnicity, household composition and potential COVID-19 exposure to older adults by working family members. We also used those data sets to assess risk factors for severe COVID cases. We analyzed CDC data collected from March 2020 through October 2020 to assess potential differences in cumulative hospitalizations by age group. We also analyzed CDC data (3) to assess distribution of long term care services by race and ethnicity. We present data for younger adults and older adults as appropriate to examine the interaction of age and race/ethnicity. We also estimate a logistic regression of the odds of delaying or not receiving needed health care by older adults to identify the extent to which different proximal indicators of inequity explain racial/ethnic health care access.

## Results

Following the WHO Health Inequity Causal Model, we examine inequities that can impact the experiences of older adults of color in the US with the COVID-19 pandemic. We start with the social context, which contributes to differential exposures, differential vulnerability, differential outcomes, and differential consequences. In a cascading manner, each of those differential levels provides a context for the patterns at the succeeding level, demonstrating complex causal process of causation for this population.

### Social Context

Table 1 shows the poverty and near-poor (100–199% FPL) rates by race/ethnicity and age. Those below the official federal poverty threshold level (FPL) are somewhat higher for younger (age 18–64) than older adults among Blacks, AI/AN, and non-Latinx Whites, but are somewhat higher for older adults among Latinxs and Asians. The proportion just above the poverty line (100–199% FPL) is higher among older adults than younger adults in all racial/ethnic groups. When examining all those with lower incomes (under 200% of the FPL), who generally will struggle to make ends meet (12), the racial/ethnic inequities are substantial: 43.1% for Blacks, 42.3% for Latinxs, and 40.6% for American Indians and Alaska Natives, vs. 24.8% for non-Latinx Whites (Table 1).

**Table 1:** Poor and near poor older adults by race and ethnicity, US 2019.

	**<99% FPL**	**100–199% FPL**	**200+ FPL**
Black^*^			
18–64	22.2	18.8	59.0
65+	20.6	22.5	56.8
**Latinx**			
18–64	16.0	21.8	62.2
65+	18.9	23.4	57.7
Asian^*^			
18–64	11.3	10.9	77.9
65+	13.6	14.8	71.5
**AI/AN∧**			
18–64	24.6	19.5	55.9
65+	18.3	24.4	57.3
White^*^			
18–64	11.4	11.3	77.3
65+	9.5	15.3	75.2

* *non-Latinx, Latinx is any race*;

∧ *AI/AN (American Indian/Alaska Native) is any mention. All age differences within race/ethnicity are statistically different at p < 0.05 and all differences from Whites by age are statistically different at p < 0.05 except for Asians ages 18–64 <99% FPL*.

*Source: US Census, American Community Survey 2019 via IPUMS (13)*.

### Differential Exposure

The extent of exposure to COVID-19 for older adults is heavily conditioned on their living arrangements. While both nursing homes and residential care facilities have lower proportions of Latinxs than the total population, older Blacks are overrepresented in nursing homes (Table 2). Nursing homes have widely reported shortages of personal protective equipment (PPE) and staff shortages, contributing to poor infection control at many homes (14). Assisted living facilities provide services to residents primarily with personal care aides who do not have infection control training and are not considered medical personnel, putting them lower on the priority list for obtaining PPE (13).

**Table 2:** Long-term care use by race and ethnicity, US 2015–16.

	**Nursing home %**	**Residential care %**	**US Population ages 65 and over %**
Latino	5.4	3.1	7.7
Non-Latinx White	75.1	81.4	78.3
Non-Latinx Black	14.3	4.1	8.7
Non-Latinx other	5.1	11.5	5.3

*Numbers may not total 100% due to rounding. Source: Harris-Kojetin L, Sengupta M, Lendon JP, Rome V, Valverde R, Caffrey C. Long-term care providers and services users in the United States, 2015–2016. National Center for Health Statistics. Vital Health Stat 3 (43). 2019. https://www.cdc.gov/nchs/data/series/sr_03/sr03_43-508.pdf*.

Over one-third of Black, and two-fifths of Latinx and Asian older adults, live in multigenerational households compared to less than one-fifth of non-Latinx Whites (Table 3). While 2% of non-Latinx White older adults who live in multigenerational households are in overcrowded living spaces, 14.5% of Asian older adults are as are 12.4% of older Latinxs (Table 3).

**Table 3:** Persons ages 65 and older in Multigeneration Households, US 2019.

	White^*^	Asian^*^	**Latinx**	Black^*^
% total living in multigenerational household	17.2	46.1	45.0	33.9
Of those living in multigenerational households, % in overcrowded housing (>1 person per room)	2.0	14.5	12.4	4.1

* *non-Latino, Latino can be any race. Source: American Community Survey 2019 via IPUMS (13)*.

The definition of essential workers varies by state, but common underlying features are the workers must work on-site to complete their jobs (remote work is not an option) and that the work is necessary for the operation of “critical infrastructure” (15). To create a proxy for potential inequities in exposures, Table 4 shows the percent of middle-aged (ages 50–64) workers in different high exposure fields who live in three generation households (i.e., likely to have an older adult in the household). Among non-Latinx White workers in all occupations ages 50–64, 5.3% live in multigenerational households, while the proportion among persons of color is two to three times higher.

**Table 4:** Workers ages 50–64, Percent living in 3-generation households by occupation, US 2018.

	**Black**	**Latinx**	**Asian**	**White**
Healthcare	13.2	14.4	10.3	5.1
Food and Agriculture	10.2	17.2	14.0	6.5
Personal care and Services	11.5	16.1	10.2	4.3
All occupations	12.3	15.9	11.2	5.3

*Source: American Community Survey 2019 via IPUMS (13)*.

### Differential Vulnerability

There is an association between the seriousness of the infection and several other factors including obesity, smoking, and a variety of chronic conditions with COVID-19 (16). Table 5 shows that while smoking declines with age, obesity and the rates of chronic conditions increase with age. Black older adults have the highest levels of vulnerability in these indicators, with Latinxs and White older adults having similar vulnerability profiles. Asian older adults have the lowest indicators of vulnerability.

**Table 5:** Risk factors for severe COVID-19, US 2018.

	**Current smoker**	**Obese**	Reports two or more chronic conditions identified as risks for severe COVID-19 (of 9^*^)
**Black#**			
18–64	16.6	18.8%	6.5
65+	13.1	25.2%	30.4
**Latinx**			
18–64	10.6	13.0%	3.1
65+	8.2	18.1%	24.4
**Asian#**			
18–64	8.4	5.1%	2.0
65+	3.9	5.5%	17.7
**White#**			
18–64	18.3	14.4%	5.3
65+	8.7	17.4%	23.1

* *Chronic conditions include: asthma, cancer, heart disease, hypertension, diabetes, emphysema, kidney disease, sickle cell, stroke (15)*.

# *non-Latinx*.

*Source: U.S. National Health Interview Survey, 2018 (17)*.

### Differential Outcomes

Table 6 shows infection rates based on Medicare claims and encounter data, separating out those with very low incomes who also qualify for Medicaid from those with higher incomes who only have Medicare. The infection case rates are 2–3 times higher for dual eligibles (Medicaid and Medicare), who are the lowest-income recipients, compared to those with only Medicare. Black, Latinx, and AI/AN dual-eligibles have similar infection rates as non-Latinx whites, while the infection rates are as much as 50% higher for persons of color compared to Whites with only Medicare. Asians show the lowest infection rates overall. In contrast to infection rates, hospitalization rates vary substantially by race/ethnicity for both income groups. Despite having similar infection rates, dual-eligible (poor) Blacks, Latinxs, and AI/ANs have higher hospitalization rates than Whites.

**Table 6:** COVID-19 infection and hospitalization rates/100,000 Medicare beneficiaries, by race, ethnicity and Medicaid eligibility, US, January 1-November 21, 2020.

	**Black**	**Latinx**	AI/AN^*^	**Asian**	**White**
**Infection rates (#/100,000)**					
Medicare + Medicaid	6,754	6,851	6,833	3,325	6,385
Medicare only	2,804	2,978	3,281	1,293	2,091
**Hospitalization rates (#/100,000)**					
Medicare + Medicaid	2,490	2,272	2,507	1,070	1,444
Medicare only	1,050	720	1,272	340	465

* *American Indian/Alaska Native*.

*Source: Center for Medicare and Medicaid Services. Preliminary Medicare COVID-19 Data Snapshot Medicare Claims and Encounter Data: Services January 1 to November 21, 2020, Received by December 18, 2020. https://www.cms.gov/research-statistics-data-systems/preliminary-medicare-covid-19-data-snapshot*.

**Figure 2:**
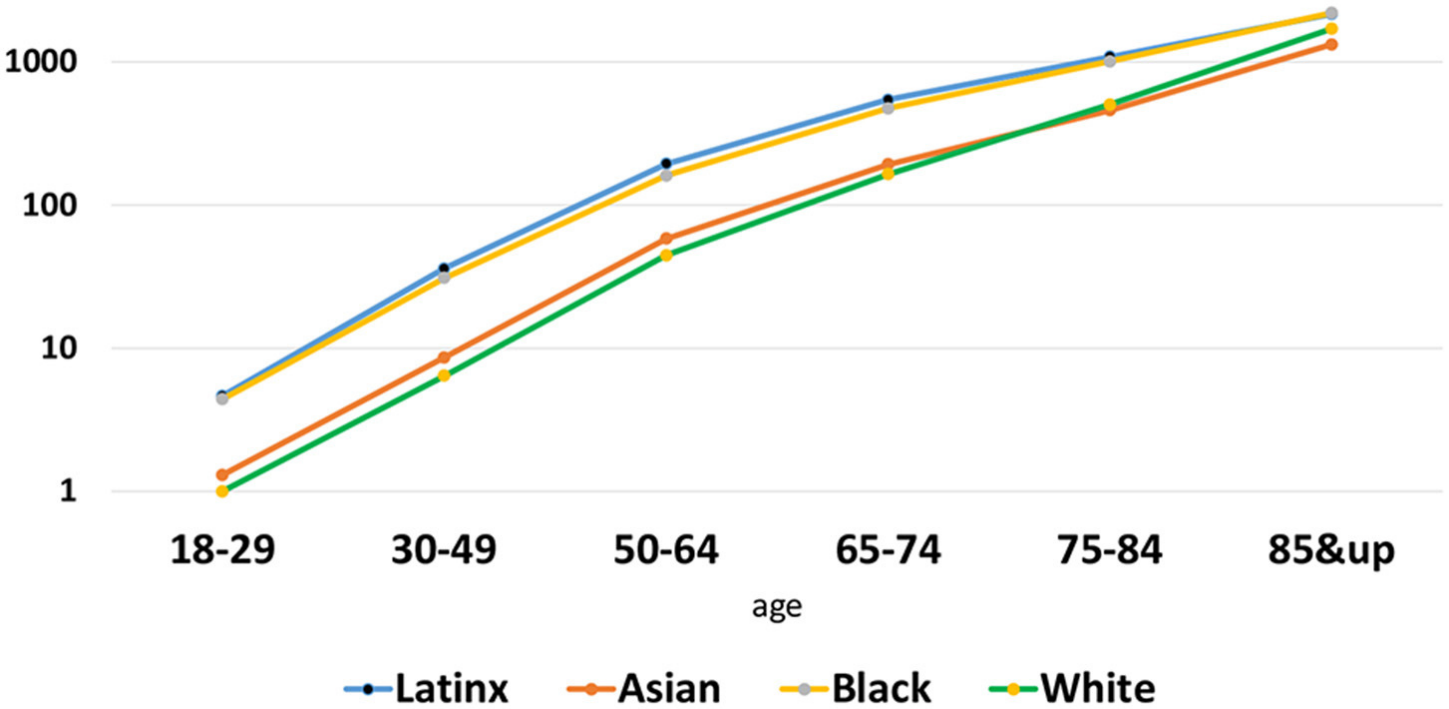
Log of Cumulative COVID-19 deaths/100,000 population, January 2021.

The most severe outcome of COVID-19, which occurs mostly among older adults, is death. The death rate increases exponentially with age, and is inequitable by race/ethnicity across all ages. Figure 2 displays deaths/100,000 population, using a log-scale so that differences among younger ages when deaths are less common and older groups where deaths are concentrated can both be seen. As of January 2021, the cumulative COVID-19 death rate nationally for non-Latinx Whites ages 18–29 is 1/100,000 (a total of 273 deaths nationwide by January 16, 2021). Blacks and Latinxs of the same ages recorded more deaths for smaller populations, making about 4/100,000 deaths. At ages 65–74, there were about 15,000 cumulative deaths nationally in each of the Black and Latinx communities, making death rates of 472/100,000 and 545/100,000 respectively. The Latinx rate is higher since the population size is smaller in that age range. The rates for Blacks and Latinxs are about three times the non-Latinx White death rate from COVID-19 of 164/100,000 population ages 65–74.

### Differential Consequences

The majority of those with COVID-19 infections survive, but the consequences of the pandemic—for both those who become infected and for those who have not—are distributed inequitably by race/ethnicity. Black and Latinx older adults are more likely to delay medical care and not get needed medical care in the past month specifically due to the COVID-19 pandemic (Table 7). Combining delay and not obtaining care, 36% of Black and 39% of Latinx older adults reported their medical care disrupted due to the pandemic, compared to 31% of White and 26% of Asian older adults.

**Table 7:** Adults ages 65 and over who delayed or did not get needed medical care past 4-weeks due to COVID-19 pandemic, US.

	**Black**	**Latinx**	**Asian**	**White**
Delayed medical care	31.5	34.8	22.5	27.9
Did not get medical care	23.9	27.1	19.2	18.4
Delayed or did not get medical care (combined)	36.3	39.5	26.1	30.7

*Source: U.S. Census, Household Pulse Survey (18)*.

In logistic regressions of delay/not get care, the pattern seen in the cross-tabulation weakens modestly when self-assessed health is added (Table 8, model 2). The Black-White difference is no longer statistically significant after controlling for self-assessed health. The odds of delay increases almost exponentially with each increment of poorer health—which would also create increased need for medical care. Adding sex (model 3) show females have a 22% greater odds of delay or not obtaining needed care, but the added variable changes the other effects little. Finally, economic need almost doubles (OR = 1.99) the odds of delay/not getting care, but Latinxs are still statistically significantly more likely to delay or not receive care (OR = 1.16). Asian older adults are 26% less likely to delay/not receive care, and other/multiple races are 2.3 times more likely to delay/not receive care. The other/multiple category is difficult to interpret since it includes AI/ANs, Pacific Islanders, and those who marked more than one race (excluding Latinx) on the survey, and cannot be further disaggregated.

**Table 8:** Logistic regression of adults ages 65 and over who delayed or did not get medical care past 4-weeks due to COVID-19 pandemic, US, December 2020.

	**Odds ratio**	**Odds ratio**	**Odds ratio**	**Odds ratio**
**Race/ethnicity: white# (ref)**				
Black#	1.29^**^	1.10	1.09	0.97
Asian#	0.79	0.75^*^	0.77^*^	0.74^*^
Other/multiple#	2.59^***^	2.60^***^	2.60^***^	2.30^***^
Latinx	1.46^***^	1.27^***^	1.28^***^	1.16^*^
**Self-assessed health: excellent (ref)**				
Very good		1.34^***^	1.33^***^	1.28^***^
Good		1.81^***^	1.81^***^	1.59^***^
Fair		3.00^***^	2.98^***^	2.31^***^
Poor		4.68^***^	4.66^***^	3.47^***^
**Gender: male (ref)**				
Female			1.22^***^	1.18^***^
Difficulty past week paying usual household expenses				1.99^***^

# *non-Latino*,

* *p < 0.05*,

** *p < 0.01*,

*** *p < 0.001*.

*Source: U.S. Census, Household Pulse Survey (18)*.

## Discussion

Our analysis of data using the World Health Organization's Health Inequity Causal Model points to multiple social determinants of health as key factors putting older adults of color at most risk of negative COVID-19 outcomes and consequences. Our findings are consistent with causal models of the effects of racism and economic inequality, and how these lead to differences in social context.

We began by noting income differences, which lead to higher proportions of older adults of color compared to Whites living with incomes under 200% of the FPL. Income is the most discussed “fundamental cause” or social determinant of health and it interacts with racism in driving intermediate social determinants of health (16). Age also plays a fundamental role as an indicator of sustained experiences of social and economic disadvantage, as a social category that brings stigma and discrimination in some contexts (12), and as an indicator of biological changes that increases the risks of chronic conditions and disabilities. Nevertheless, older adults are often portrayed in policy discussions as well-off, enjoying paid-off mortgages for their homes, good pensions, and Medicare with supplemental coverage to pay most of their health costs. While this does accurately describe a segment of the older adult population, there are many older adults—more commonly older adults of color—who have lower educational attainments, incomes that are inadequate to pay basic costs of living, and poor health with inadequate access to health care (16). Low incomes are created in part by historic patterns of racism in education, employment, and housing throughout the life course, creating cumulative disadvantages (19) for older adults of color in years of education, rates of private pensions and other sources of income, and inadequate housing in segregated and service-poor neighborhoods (20). This set of inequitable contexts contributes to the next dimension, differential exposures.

We show how older adults of color experience differential exposure to the virus due to their living arrangements, including overcrowded housing or living in densely populated settings. Those in institutional settings have the highest risk of exposures based on their sustained interaction indoors to multiple potentially infected persons (facility staff) where the older adults have little agency to reduce risks. For those living in the community, older adults in households with essential workers, who are at the highest risk of community exposure, will experience the greatest risk of exposure. That exposure risk will be heightened by factors such as overcrowded housing and living in densely populated buildings and neighborhoods that contain further opportunities for exposure to the virus. Nursing homes have widely reported shortages of PPE and staff shortages, contributing to poor infection control at many homes (14). Assisted living facilities provide services to residents primarily with personal care aides who do not have infection control training and are not considered medical personnel, putting them lower on the priority list for obtaining PPE (13).

Another factor that increases the risk of exposure even more is that older adults of color who live in multigenerational households are much more likely to live in overcrowded housing, defined as more than one person per room (21). To the extent that younger adults are more likely to be in the labor force and to the extent that workers of color are less likely to be able to work from home, older adults in multigenerational households are likely to increase their risk of being exposed to others who carry the virus. The common pattern of older adults of color living in multigenerational households also increases their chances of living with others who are frontline essential workers. The rates of three generational households varies somewhat by occupation, but the inequity between racial/ethnic groups remains large. In addition, workers of color are more concentrated than non-Latinx Whites in frontline occupations and are further concentrated in low-waged occupations where personal protective equipment is least available (9). In sum, there is likely a differential exposure to COVID-19 by older adults of color compared to non-Latinx Whites, driven by inequities created by economics and racism in housing quality (institutionalization, community overcrowding) and labor force segmentation by race/ethnicity.

Older adults also experience differential vulnerability to the effects of the virus, due to their risks for chronic conditions that increases with age, and different groups can have different responses to the same exposures based on their vulnerability to the disease. We note the increased impacts of infection, hospitalization, and death rates in communities of color. The higher rates of vulnerability for older Blacks have been attributed to a number of different social determinants of health. Racism is a fundamental cause, contributing to increased stress as well as lower incomes, poorer access to health care, and residential segregation in areas with inadequate infrastructure related to healthy nutrition, physical activity, and health care (22, 23). Each of those, in turn, are associated with each of the conditions and behaviors that are noted above as increasing the impact of severe illness with COVID-19 (24, 25). The similar vulnerability (prevalence of complicating health conditions) for Latinxs and non-Latinx Whites reflects the well-documented epidemiological paradox of Latinxs as a group having lower education, income, worse working conditions, and poorer housing than non-Latino whites, yet having better than expected mortality and disease profiles. Much of the health advantage can be attributed to the high proportion of the Latinx population that is immigrant (and is even higher among Asians) who arrive in better health, although much of the advantage in risk factors declines with time in the US (17, 18, 26). Though this is being challenged (27, 28).

We demonstrate multiple differential outcomes of the pandemic among Black and Latinx older adults. For those who contract COVID-19, there are differential health outcomes. Black older adults in particular have higher rates of vulnerabilities for severe COVID-19 outcomes. There is a higher infection rate among older Blacks, American Indians/Alaska Natives, and Latinxs than among non-Latinxs Whites. This is partially, but not fully explained by economic differences. The racial/ethnic inequities remain (except for Asians) across groups compared to Whites in hospitalization rates. While there is no literature or models directly predicting this, it is possible that the higher rate of mask-wearing by Asian Americans than others contributes to the lower rates of spread (29), despite higher rates of co-residence and other exposure risk factors for older adults. This is not surprising given the inequities in vulnerabilities among older adults of color, which lead to more severe conditions. It is also possible that persons of color delay seeking treatment of the disease due to health system barriers they face, as has been found with other acute health conditions (30). This results in people of color presenting with more severe stages of the infection and requiring more inpatient than outpatient treatment. Delays and avoiding needed health care among older adults is particularly worrisome given the high levels of chronic conditions and other health issues that are distributed inequitably to start with among the population. Although these racial/ethnic differences are driven in part by differences in health status and economic barriers, there are multiple possible causes of the remaining inequities, including having unequal access to public transportation, feeling unsafe on public transportation, unequal access to telehealth, being uncomfortable with telehealth, fears of contracting COVID-19 at the doctor's office, new childcare responsibilities for grandchildren studying remotely from home, and other changes brought on by the COVID-19 epidemic that impact communities of color inequitably due to racism and economic disadvantages. The apparently advantaged situation of Asian American older adults deserves additional research to understand; it is possible the bimodal distribution of economic and social resources among Asian American older adults that varies by country of origin may be obscuring inequities within that group that merit further attention (31, 32). Unfortunately, almost all COVID-19 and much other health data fail to disaggregate any of the standard racial/ethnic designations that would be needed for further analysis.

Death is the most severe outcome of COVID-19. The death rate increases exponentially with age, and are inequitable by race/ethnicity across all ages. These disparities are likely driven by the differential exposures and vulnerabilities described above, compounded by differences in access to and quality of health care. Despite the advantaged infection and hospitalization rates noted above, the death rate for Asians closely tracks the White rate, suggesting there are factors we have not identified here that are converting a higher proportion of infections and hospitalizations to deaths among Asian Americans than non-Latinx Whites. In addition, we note differences between older adults and younger adults in each group since older adults have the highest death rates from COVID-19 and often face higher rates of causal risks than younger adults, combined with their life-long exposures to disadvantage which can put them in more disadvantaged conditions than younger adults.

A social determinants of health approach points to the systemic causes of these inequities that need to be addressed over time, but the harms of those inequities are being experienced now by older adults in communities of color during the COVID-19 epidemic. The multilayered WHO model highlights the differences in risks and outcomes, and how the cascade of socially determined risks leads to adverse outcomes in some communities and among certain individuals. This is useful in identifying what can be done now to address the health disparities that the COVID-19 is revealing. Many of these social determinants of health can be addressed through the implementation of health policies that have a broad equity focus like calls for the establishment of a universal food income (33) and expanded investments in home-based care. Adding a human rights frame to a public health perspective to address the pressing issues raised by the current pandemic would facilitate the development of health care policies that are inclusive of all members of society, especially the most vulnerable. Doing this now would also facilitate addressing other pressing health disparities and decrease the negative impacts of systemic neglect of particular communities moving forward.

We have the opportunity now to refocus discussions on what is good for the health of all people living in our country, and to move beyond a simple analysis of individual-level risk factors (socio-economic status, race/ethnicity, or age) that may contribute to disparities. As researchers and academics, we need to ensure that the multiple calls for equity-focused solutions and systemic responses to the issues raised by the COVID-19 pandemic are operationalized and implemented (34, 35). For example, the discussions around the distribution of the COVID-19 vaccine are often centered on the logistics of large-scale distribution (i.e., large, drive in sites), with little attention to addressing the need for community-based distribution models (for those who may lack transportation or are unable to wait long hours in a car), or the accessibility problems raised by using mostly web-based registrations, or to the social realities that make some older adults more vulnerable than others to getting the disease (eg. household composition), or to the hesitancy of individuals in communities of color getting the vaccine once it is made available (e.g., distrust of the medical community). Investing the time and effort to address the multiple dimensions of interacting and intersecting causes of health inequities, and grounding solutions in community-based and community-specific needs, could potentially create an infrastructure for the dissemination of other health programs (including other vaccines) in the future. Recognizing and elevating the important work of *promotores* (or community health workers), home care providers, and caregivers in general, who are managing the fragmentation issues in our health care systems, also sheds light on the needs of older adults of color. In short, addressing the multiple social determinants of health that contribute to the negative outcomes of COVID-19 on older adults of color will help facilitate health equity now. Yet, there is a still a need for long-term health equity work to improve equity in housing, education, labor force protections, and basic incomes in order to ensure health equity for all in the future.

## Data Availability Statement

Publicly available datasets were analyzed in this study. This data can be found at: URLs provided in the citations to the data sources.

## Author Contributions

All authors listed have made a substantial, direct and intellectual contribution to the work, and approved it for publication.

## Conflict of Interest

The authors declare that the research was conducted in the absence of any commercial or financial relationships that could be construed as a potential conflict of interest.
